# TTBK2‐Driven Ciliogenesis Is Required for Intrinsic Neuronal Regeneration After Spinal Cord Injury

**DOI:** 10.1002/cns.70763

**Published:** 2026-01-24

**Authors:** Renfeng Zhang, Su Pan, Zhenwei Tian, Haorui Du, Jintao Wang, Xiaoyu Yang, Zhiping Qi

**Affiliations:** ^1^ Department of Orthopedic Surgery Second Hosp Jilin University Changchun China; ^2^ Department of Emergency & Critical Care Second Hosp Jilin University Changchun China

**Keywords:** axonal regeneration, primary cilia, spinal cord injury, TTBK2

## Abstract

**Background:**

The primary cilium (PC) is a pivotal organelle for neuronal signaling and development, while tau tubulin kinase 2 (TTBK2) is a key initiator of ciliogenesis. However, the role of TTBK2 in spinal neurons during spinal cord injury (SCI) and subsequent neural repair remains unclear.

**Methods:**

We manipulated TTBK2 expression in spinal neurons using adenovirus‐mediated overexpression and knockdown in vitro. Transcriptomic profiling (RNA‐sequencing) and RT‐qPCR were employed to explore the potential regulatory pathways at the molecular level. In vivo, *Ttbk2* ^fl/fl^‐Rosa‐CreERT2^+/−^ conditional knockout mice were subjected to a spinal cord hemisection model. Behavioral assays, immunofluorescence, and biotinylated dextran amine (BDA) tracing were conducted to assess neuronal survival, axonal regeneration, and circuit reorganization.

**Results:**

Here, we demonstrated that intact activity of TTBK2 in PC promoted neural survival, axonal regeneration, and neural circuit remodeling. However, loss of TTBK2 impaired PC function and hindered recovery after SCI.

**Conclusions:**

These findings extend the role of PCs beyond neurodevelopment, demonstrating that the TTBK2–PC functions as an endogenous repair mechanism after SCI. Targeting this pathway may provide novel therapeutic strategies for enhancing neural regeneration.

## Graphical abstract

TTBK2‐dependent ciliogenesis is required for intrinsic neuronal regeneration after spinal cord injury. Loss of TTBK2 disrupts primary cilium integrity, attenuates SHH signaling, and impairs axonal regrowth. Restoring SHH activity partially rescues neuronal structural deficits, highlighting the TTBK2/cilium–SHH as a therapeutic target.

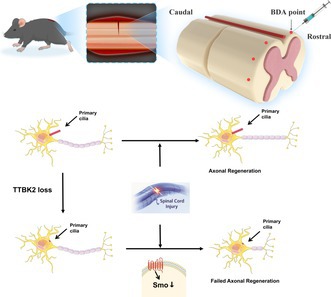

## 1 Introduction

SCI is defined as damage to the spinal cord tissue caused by various factors, resulting in axonal disconnection and neuronal death. These lead to motor, sensory, and neurological dysfunctions below the lesion location and usually cause permanent impairment. However, curative treatments are lacking [1]. The regenerative ability of neurons after SCI is limited, primarily owing to the pronounced inhibitory signals in the injured microenvironment [2]. Nevertheless, when the intrinsic regenerative ability of neurons is not activated, reducing inhibitory signals in the microenvironment induces insufficient neuronal regeneration [3]. Therefore, promoting the intrinsic regenerative ability of neurons is crucial in injury repair.

PCs are non‐motile, microtubule‐based cellular antennae that are predominant on the surface of quiescent vertebrate cells [4, 5]. Their core structure is anchored by a basal body derived from the mother centriole and extends a “9 + 0” microtubule doublet axoneme lacking a central pair. The axoneme is covered by a specialized ciliary membrane enriched with components, such as G protein‐coupled receptors (GPCRs) and ion channels [6, 7]. The transition zone between the basal body and the axoneme represents a selective “molecular gate” that strictly regulates molecular entry and exit [8]. Intraflagellar transport (IFT) within the cilium, driven by kinesin‐2 (anterograde) and dynein‐2 (retrograde), enables the bidirectional transport of signaling proteins and structural components [9]. In the nervous system, the PC plays a particularly prominent role. During hippocampal neurogenesis in mice, the cilium orchestrates the SHH signaling cascade to regulate the differentiation of neural precursor cells into distinct neuronal subtypes [10, 11]. The ordered localization and dynamic trafficking of signaling molecules—such as Smoothened (Smo), Patched (Ptch1), and Gli transcription factors—within the cilium constitute core mechanisms regulating cell fates [12]. Growing evidence establishes the PC as a central signaling hub that integrates multiple key pathways. For example, in Hedgehog signaling, activated Smo receptors translocate to the ciliary membrane and regulate Gli transcription factors to modulate hippocampal neurogenesis [7]; non‐canonical Wnt signaling guides neuronal differentiation [13]; platelet‐derived growth factor receptor α (PDGFR‐α) mediates responses to growth factors [14]. Additionally, PCs participate in controlling the direction, speed, and final positioning of neuronal migration [15].

TTBK2 is a serine/threonine kinase that plays a key regulatory role in the initiation of PC assembly [16]. TTBK2 phosphorylates key basal body substrates, such as CEP164, recruiting downstream effectors (e.g., CEP83 and CEP89) to promote the formation of distal appendages and facilitating ciliary membrane docking and axoneme elongation [17, 18, 19]. In addition, TTBK2 maintains the stability of the proteins required for ciliogenesis by suppressing autophagic degradation [20, 21]. Studies have shown that loss‐of‐function mutations in TTBK2 disrupt cilium formation, impair the connectivity and survival of cerebellar Purkinje neurons, and cause spinocerebellar ataxia, underscoring the critical link between TTBK2 dysfunction and central nervous system disorders [16, 22].

Although the regulatory roles of TTBK2 and PC in CNS development and neuronal networks have been established, their functions in CNS injuries, particularly SCI, remain largely unexplored. Therefore, we aimed to investigate the role of TTBK2‐mediated PC signaling in neural repair and regeneration after SCI and the specific endogenous repair mechanisms it possibly influences.

We downregulated and overexpressed TTBK2 in spinal neurons via in vitro transfection and assessed their effects on axonal regeneration. Using transcriptomic and proteomic profiling, we identified and validated downstream pathways and key targets of the TTBK2/PC. Furthermore, we established a conditional Ttbk2‐knockout mouse model with spinal hemisection to investigate the functional and mechanistic contributions of TTBK2/PC signaling to functional recovery and neural repair after SCI. Our study extends the relevance of PCs beyond neurodevelopment, reveals the essential regulatory functions of the TTBK2/PC in endogenous repair after SCI, and provides a rationale for potential therapeutic strategies targeting neuroregeneration in SCI.

## 2 Materials and Methods

### 2.1 Spinal Neuron Cell Culture

Neuronal cells were isolated from the spinal cord of fetal mice on the 15th day of gestation. Cells were cultured at 1.5 × 10^6^/well (6‐well plate) for Western blot, and neuronal cells were grown in a U‐Plate 24‐Well Black (Ibidi, Germany) for immunofluorescence assays. The neuronal growth medium was DMEM (Gibco, USA), supplemented with fetal bovine serum (FBS) (Gibco, USA). The neuronal media included a neurobasal medium (Gibco, USA), 2% B27 neural supplement (Gibco‐Invitrogen, USA), 1 mM L‐glutamine solution (Sigma‐Aldrich, G7513, USA), and 100 ng/mL penicillin–streptomycin (Gibco‐Invitrogen, USA). Neuronal cell culture medium was changed every 2 and a half days, and subsequent experiments were performed on Day 5 to identify neuronal cells using immunofluorescence assays.

### 2.2 Ttbk2^fl/fl^‐Rosa‐Cre ERT‐Knockout Mouse Construction

*Ttbk2* ^fl/fl^ mice were constructed using the Cre‐Loxp system (Shanghai Model Organisms Center Inc., China), followed by the construction of Rosa‐Cre ERT^+/−^mice, and finally, *Ttbk2* ^fl/fl^‐Rosa‐Cre ERT^+/−^ mice were obtained. The mice were genotyped using agarose gel electrophoresis 14 days after birth. The primers used are listed in Table 1. At 4 weeks, *Ttbk2* knockout was induced in Rosa‐Cre ERT^+/−^ mice through the injection of tamoxifen (Sigma‐Aldrich, 10540‐29‐1, USA), which was dissolved in corn oil (Beyotime, China) at 20 mg/mL. Each mouse was injected at 120 mg/kg, every other day, receiving a total of five injections. Subsequently, the mice were left to rest for 1 week. Subsequently, knockout identification and subsequent experiments were performed.

**TABLE 1:** Primer sequence.

		Primer sequence
TTBK2 Wild type	F	TCAGATTCTTTTATAGGGGACACA
	R	TAAAGGCCACTCAATGCTCACTAA
TTBK2 Mutant	F	TCCGGGCTGCCACGACCAA
	R	GGCGCGGCAACACCATTTTT
TTKB2	F	ACTCCAAGTTCTTCCCCTTTTC
	R	GTTTTCTCATTCTCCCATCGTC
KIF3A	F	TGAAGTCGACCTTTCCCACG
	R	CAGGCTTTGCAGAACGCTTT
Smo	F	TTCACACTGGCCACCTTTGT
	R	TCCCCAAATCTCATGGTGCC
Gli1	F	CACACCTGCTCAGCACTACA
	R	AAGACCTCCCATCCGATCCA
SUFU	F	ACTACGGACAGTGCCCATTG
	R	CAGGTGCGGATCGATCTCAA
akt1	F	CGCTTCTATGGTGCGGAGAT
	R	GTTCTCCAGCTTCAGGTCCC
mtor	F	AAGGCCTGATGGGATTTGGG
	R	GGGGCAGCAGGTTAAGGATT
pten	F	ACAATTCCCAGTCAGAGGCG
	R	CAGTGCCACGGGTCTGTAAT
Calmodulin	F	GAAGATGTGCGACCCTGGAA
	R	GAGGGTTCAGGATGGTGGTG
β‐Actin	F	TACTGCTCTGGCTCCTAGCA
	R	CGGACTCATCGTACTCCTGC

### 2.3 Ttbk2^fl/fl^‐AAV9‐Hsyn‐Cre Knockout Mouse Construction

In this study, spinal cord targeted delivery of the AAV9‐hSyn‐Cre virus was used to generate a neuron‐specific *Ttbk2* conditional knockout in *Ttbk2* ^fl/fl^ mice. Under stereotaxic guidance, the virus (2 × 10^13^ vg/mL) was microinjected into the dorsal horn gray matter at spinal levels T9 and T11, with two unilateral injection sites per segment and an injection volume of 1 μL per site. The experimental group (AAV‐Cre) received AAV9‐hSyn‐Cre, whereas the control group was administered an equal volume and titer of AAV9‐hSyn (NC) (Hunan Fenghui Biotechnology Co. Ltd). The hSyn promoter, derived from the human SYN1 gene, drives neuron‐specific expression of Cre recombinase and thus mediates excision of the floxed Ttbk2 fragment [23, 24]. 2 weeks after viral delivery, spinal cord tissue from the injection segments was harvested, and Ttbk2 knockout efficiency was assessed at both the mRNA and protein levels using qRT‐PCR and Western blot analysis.

### 2.4 Establishment of Spinal Cord T10 Lateral Hemisection Injury Model

Female mice were divided into *Ttbk2* ^fl/fl^‐Cre ERT^−/−^(WT), *Ttbk2* ^fl/fl^‐Cre ERT^−/−^‐SCI (WT‐SCI), *Ttbk2* ^fl/fl^‐Cre ERT^+/−^(*Ttbk2* ^fl/fl^), and *Ttbk2* ^fl/fl^‐Cre ERT^+/−^‐SCI (*Ttbk2* ^fl/fl^‐SCI) groups, which were treated with tamoxifen. Anesthesia was performed using 1.25% tribromoethanol intraperitoneal injection (Rhawn, China, 30 mL/kg). The skin above and below the level of T10 of the mice was removed using depilatory creams, and the spinal cord was exposed using scissors and forceps. The right side of the spinal cord of the mice was thoroughly clipped with the dorsal central blood vessels of the spinal cord as the boundary. After hemorrhage was stopped sufficiently, the muscular layer and skin were individually closed and bandaged with gauze.

### 2.5 Immunostaining

Cells were fixed with 4% paraformaldehyde for 20 min and subsequently washed thrice with phosphate‐buffered solution (PBS) (Gibco, USA), blocked with goat serum (Sigma‐Aldrich, USA) containing 0.2% Triton X‐100 + 10% NGS for 30 min, rewashed thrice with PBS, and incubated with primary antibodies for 24 h at 4°C. Subsequently, the cells were washed thrice with PBS and incubated with secondary antibodies for 40 min. The nuclei were stained with DAPI and blocked with an anti‐fluorescence quenching blocker (Beyotime, China).

All primary and secondary antibodies were diluted in PBST containing 10% goat serum. The following antibodies were used: rabbit anti‐beta III Tubulin (TUJ1) (Abcam, ab52623, 1:500), mouse monoclonal antibody to ACIII (AC3) (Encorbio, MCA‐1A12, 1:1000), rabbit anti‐ARL13B (Abcam, ab136648, 1:1000), chicken anti‐MAP2 (MAP2) (Abcam, ab5392, 1:1000), goat anti‐rabbit IgG H&L (FITC) (Abcam, ab150077, 1:5000), and goat anti‐chicken IgY H&L (Alexa Fluor 555) (Abcam, ab150170, 1:2000). Cellular staining was observed using a laser confocal microscope (FV3000, Olympus, Japan), and the staining results were semi‐quantitatively analyzed using Fiji software.

### 2.6 Adenoviral Infection of Neurons

The cells were divided into five groups: NC, shKIF3A, shTTBK2, overexpress TTBK2 (TTBK2‐OE), and shKIF3A + TTBK2‐OE. Cells were seeded on the plate on Day 5 and infected with adenoviruses. Using the 1/2‐volume infection method, half of the medium in the original well plate was removed, and the corresponding viral solution was added at a multiplicity of infection of 100 [25]. Adv5‐MIR30 (shTTBK2, NM_001024856), Adv5‐MIR30 (shKIF3A, NM_001290805), and pDC316‐mCMV (mTTBK2, NM_001024856) sequences of the target genes are presented in Table 1. The cells were transfected with plasmids to silence KIF3A, silence TTBK2, overexpress TTBK2, or combine KIF3A silencing with TTBK2 overexpression, and negative plasmids were added to the negative control group. After 8 h of infection, the virus‐shouting medium was discarded and replaced with the neuronal medium for further cultivation. The infection efficiency was determined using qRT‐PCR.

### 2.7 qRT‐PCR

Total RNA was extracted using the TRIzol reagent (Invitrogen, USA) at 48 h of infection, using the TRIzol Reagent kit (Tiangen, DP405) according to the manufacturer's instructions, and the concentration and purity of total RNA were determined using a Nanodrop spectrophotometer (M200, Tecan, Switzerland). Three biological replicates from each group were evaluated in this manner. RNA samples were reverse‐transcribed into cDNA using the PrimeScript reagent kit (Roche, Switzerland) for subsequent qPCR. The primer sequences for the target genes are listed in Table 1. *Actin* was used as an internal reference gene.

### 2.8 RNA‐Seq

RNA‐seq libraries were generated and sequenced by Beijing Novogene Technology. The reference genome and annotation files were obtained from NCBI, Uniprot, ENSEMBL, GO, and KEGG [26]. Using HISAT2 (v2.2.1) [27], we built a genome index and performed splice‐aware alignment of paired‐end reads, leveraging gene models for enhanced accuracy. We conducted differential expression analysis using DESeq2 (v1.42.0) [28], applying a negative binomial model. Genes with an adjusted *p*‐value (Benjamini‐Hochberg; *p*adj < 0.05) and |log2(FoldChange)| ≥ 1 were deemed significant.

### 2.9 Western Blotting and Protein Quantification

After 48 h of infection, total cellular proteins were extracted, the cells were washed with PBS and lysed using RIPA (Beyotime, China, P0013C) containing PMSF (Beyotime, China, P0012). Protein concentration was determined using BCA (Beyotime, China, P0009) and a microplate reader (Tecan M200 Infinite Pro). Subsequently, a loading buffer was added, with a protein concentration of 2 μg/μL for each sample. The protein was denatured for 5 min at 95°C. Total protein (20 μg) was added to a 6%–12% sodium dodecyl sulfate‐polyacrylamide gel electrophoresis (SDS‐PAGE) gel and electrophoresed at 200 V in an SDS buffer for 1 h. Polyvinylidene fluoride (PVDF) membranes (Millipore) are activated in anhydrous methanol, then proteins were transferred to the PVDF membrane via running for 1 h at 80 V and 4°C. Blocked with 5% skim milk powder for 1 h and incubated with the primary antibody on a shaker overnight at 4°C. Subsequently, the membrane was washed thrice with PBS‐T and incubated with secondary antibodies for 1 h on a shaker at 24°C.

All primary and secondary antibodies were diluted using an antibody diluent (Beyotime, China, P0023). The following antibodies were used: rabbit anti‐MAP2 (MAP2) (Abcam, ab32454, 1:1000), rabbit anti‐NogoA (Abcam, ab62024, 1:500), rabbit anti‐Nogo‐receptor (ab184556, 1:10,000), rabbit anti‐smoothened antibody (Abcam, ab236465, 1:200), mouse anti‐GLI‐1/GLI1 (C‐1) (santa sc‐515751, 1:500), RhoA (67B9) rabbit mAb (Cell Signaling, 2117, 1:1000), chicken anti‐MAP2 (MAP2) (Abcam, ab5392, 1:1000), rabbit anti‐PSD95 (Cell Signaling, 3450, 1:400), goat anti‐rabbit IgG H&L (HRP) (Abcam, ab6721, 1:10,000), B‐actin, goat anti‐chicken lgY (H + L) secondary antibody, and HRP (Thermo Fisher Scientific, A16054, 1:1000).

Images were captured using a ChemiDoc Touch imaging system (Bio‐Rad), and the protein bands were quantified using the Fiji software.

### 2.10 BDA Tracing

In the mouse model, a dorsal skin incision was made 3 cm above the injury site at approximately the T4 level to fully expose the spinal cord, and intrathecal injections were performed using a 1‐μL microinjector (USA), 1 week after the injury model is completed. With five transverse sites at T4, 1 μL was injected at each site, totaling 5 μL of BDA solution (Thermo Fisher Scientific, D1956) at an injection rate of 5 min/μL. Subsequently, the muscle and skin layers were sutured layer‐by‐layer and bandaged using gauze. After the mice were awakened, they were transferred to a mice cage.

### 2.11 Assessment of Motor Function Recovery in Mice

Motor function recovery after SCI was assessed in each group using the Basso Mouse Scale (BMS) [29]. Each group of mice was assessed once on Days 3, 7, 14, and 28, and all mice were analyzed by two independent reviewers who were blinded to the treatment of the animals. On Day 14, all mice were placed in a black box and walked on a transparent runway using colorful paws. The footprint results were collected, several parameters were obtained to assess behavioral status, and recovery from injury was assessed based on the step length (SL) of the right hind limb versus the step width (SF) between the right and left hind limbs.

### 2.12 Immunohistochemistry

After 14 days, anesthesia was administered to the mice through the intraperitoneal injection of 1.25% tribromoethanol, the spinal cord of each mouse was perfused through cardiac perfusion with saline and 4% paraformaldehyde, and the tissues were fixed with 4% paraformaldehyde for 24 h. The samples were dehydrated using a 25% sucrose solution. The spinal cord was transversely cut into 3‐μm frozen sections, which were stained with LFB (Rousseau Fast Blue) or HE and observed under a light microscope. Immunofluorescence staining was performed as follows: frozen sections were hydrated after acclimation to room temperature, soaked in PBST for 10 min, and rinsed twice with PBS; the sections were placed in a sodium citrate repair solution, heated to 95°C in a water bath for 15 min, allowed to cool to room temperature, washed with PBS, treated with drops of blocking solution, and incubated at room temperature in a wet box for 1 h. Drops of primary antibody were added overnight for 4 h, and the sections were removed and equilibrated to room temperature. The cells were incubated at 24°C with secondary antibodies for 1 h, followed by the DAPI staining of the cell nuclei.

All primary and secondary antibodies were diluted with antibody diluent (Beyotime, China). The antibodies were rabbit anti‐GFAP (Abcam, ab7260, 1:5000) mouse anti‐beta III Tubulin (TUJ1) (Proteintech, 66375‐1‐Ig, 1:400), chicken anti‐MAP2 (MAP2) (Abcam, ab5392, 1:1000), rabbit anti‐Calb (Abcam, ab108404, 1:150), mouse monoclonal antibody to ACIII (AC3) (Encorbio, MCA‐1A12, 1:1000), chicken anti‐choline acetyltransferase antibody (ChAT) (Sigma‐Aldrich, AB15468, 1:1000), rabbit anti‐TTBK2 (Sigma‐Aldrich, AB805274, 1:1000), rabbit anti‐neurofilament‐H (NF200) (Cell Signaling, 30564, 1:400), mouse monoclonal [Rat‐401] to nestin‐neural stem cell marker (Nestin) (Abcam, ab6142, 1:1000), rabbit anti‐PSD95 (Cell Signaling, 3450, 1:400), chicken anti‐GAP43 polyclonal antibody (Thermo Fisher Scientific, PA5‐95660, 1:500), goat anti‐chicken secondary antibody goat anti‐chicken IgY H&L (Alexa Fluor 555) (Abcam, ab150170, 1:2000), goat anti‐rabbit IgG (H + L) (Alexa Fluor 647) (Beyotime, A0468 1:200), goat anti‐rabbit IgG (H + L) (Alexa Fluor 350) (Beyotime, A0408, 1:200), and goat anti‐mouse IgG (H + L) (Alexa Fluor 647) (Beyotime, A0473, 1:200). All sections were visualized under a confocal laser‐scanning microscope.

### 2.13 TEM

Fresh tissues were identified for the sampling section, and the samples required for observation were cut into small pieces of approximately 1 mm^2^. The samples were fixed with 2.5% glutaraldehyde for 2 h at 4°C. Subsequently, 0.1 M acid‐rinsing solution was used to rinse the samples three times (15 min for each rinsing). Next, the samples were fixed in 1% silver acid for 4 h at 4°C. Furthermore, the samples underwent dehydration through an alcohol gradient, with each step lasting 40 min. Similarly, we treated the samples with 100% epoxy propylene three times (30 min for each treatment). For embedding, the samples were placed in the embedding solution at room temperature overnight, followed by replacement with pure embedding solution for 3–4 h at room temperature. The samples were then transferred to a 60°C oven for 48 h to allow the complete polymerization of the resin. After polymerization, the embedded blocks were removed and prepared for ultra‐thin sectioning. Sections of 70‐nm thickness were cut and collected on copper mesh grids. The sections were stained with 3% uranyl acetate in saturated alcohol solution for 8 min, followed by washing with 70% alcohol (three times) and ultrapure water (three times). Additionally, the sections were stained with 2.7% lead citrate solution for 8 min and washed with ultrapure water (three times). After slight drying with filter paper, the sections were observed and photographed under a transmission electron microscope.

### 2.14 Pharmacological Modulation of the SHH Pathway

To assess the necessity and sufficiency of SHH signaling in SCI repair, we performed intrathecal pharmacological modulation of the SHH pathway in wild‐type and *Ttbk2* ^fl/fl^ mice. Animals were allocated into five groups for pharmacological analysis: WT, WT‐SCI, WT‐SCI + Cyclopamine, *Ttbk2* ^fl/fl^‐SCI, *Ttbk2* ^fl/fl^‐SCI + SAG. Cyclopamine (Selleckchem, S1146) was dissolved according to the manufacturer's instructions and administered at 50 mg/kg via intrathecal injection starting on the day of injury and continuing once daily for 5 consecutive days. For SHH pathway activation, SAG (Selleckchem, S6384) was dissolved in 10% DMSO and administered intrathecally at 10 mg/kg, beginning on the day of injury and continuing for 5 consecutive days. At the designated endpoints, spinal cord tissues encompassing the lesion core and adjacent segments were collected for Western blot analysis of MAP2 and SHH‐related markers. All drug doses were selected based on previous in vivo studies demonstrating effective SHH pathway modulation in rodent CNS tissues [30].

### 2.15 Quantification and Statistical Analysis

All quantitative data were analyzed using GraphPad Prism 5.0 software (GraphPad Software, USA) and expressed as mean ± standard deviation. One‐way analysis of variance was used for more than two variables, and a *t*‐test was used for two groups. One‐way ANOVA followed by post Dunnett's or Tukey's multiple comparisons tests was employed for datasets with one independent factor across multiple groups. Whereas, two‐way ANOVA followed by Sidak's multiple comparisons test was used for datasets involving two independent factors. *p < 0.05 indicated statistical significance. **p < 0.01 and ***p < 0.001 implied high statistical significance.

## 3 Results

### 3.1 TTBK2 Regulates Spinal Neuronal Axon Growth via PCs

**FIGURE 1:**
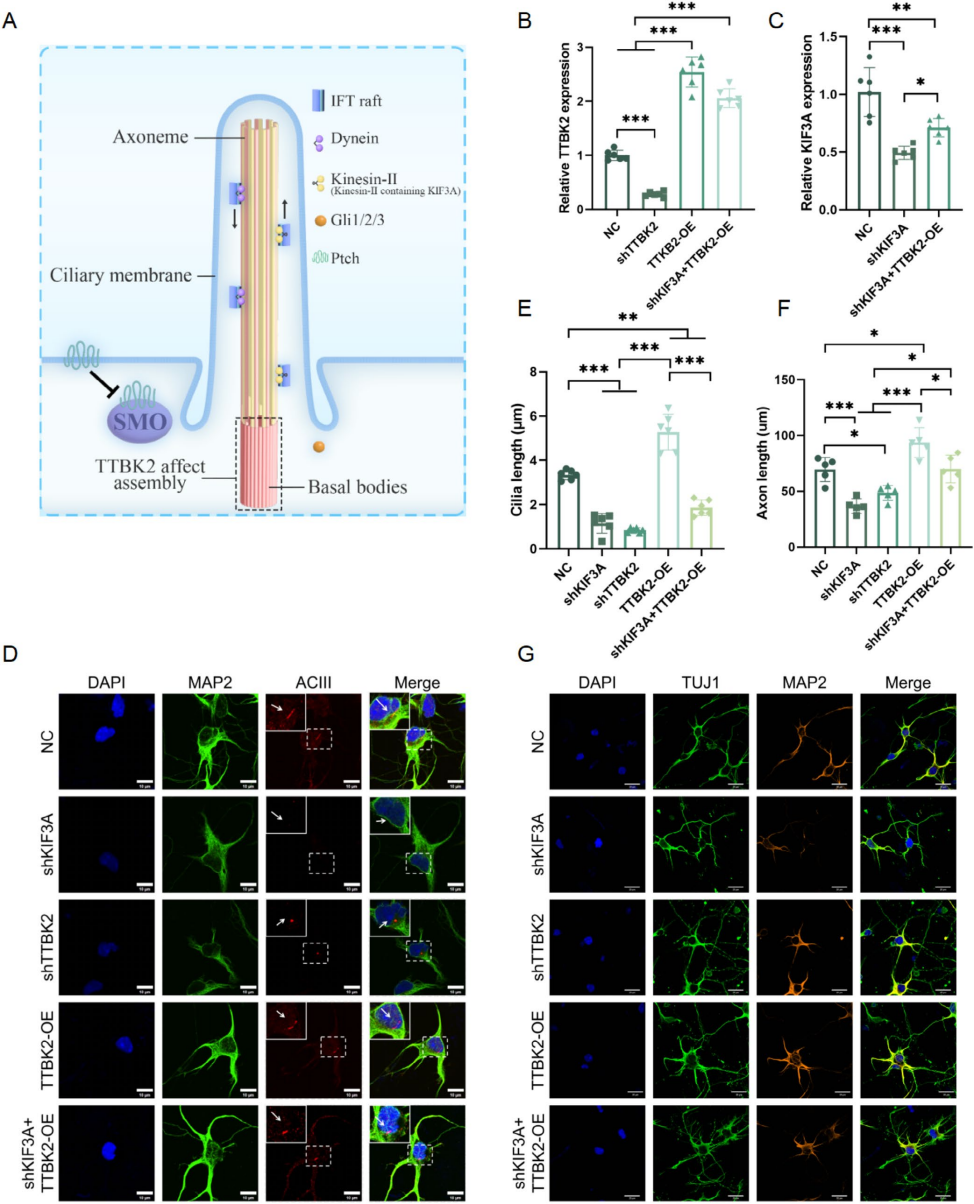
TTBK2 regulates primary cilium formation and axonal growth in spinal neurons. (A) Schematic diagram of the primary cilium. Kinesin‐2 comprises KIF3A; TTBK2 is related to the formation of basal bodies. (B, C) Quantitative RT‐qPCR analysis showing efficient knockdown or overexpression of KIF3A and TTBK2 in spinal neurons via adenoviral infection (*n* = 6 from 3 independent experiments). (D, E) Representative immunofluorescence images of spinal neurons labeled with MAP2 (green), ACIII (red), and DAPI (blue) in five experimental groups: NC, shKIF3A, shTTBK2, TTBK2‐OE, and shKIF3A + TTBK2‐OE. White arrows indicate PCs. Compared with that in NC, the cilium length was significantly reduced in the shKIF3A, shTTBK2, and shKIF3A + TTBK2‐OE groups, while TTBK2‐OE overexpression led to elongated cilia (*n* = 6 from 3 independent experiments). Scale bars, 20 μm. (F, G) Representative images showing immunolabeling of TUJ1 (green, axons), MAP2 (orange, dendrites), and DAPI (blue, nuclei). Axonal morphology and length were assessed across five groups. KIF3A or TTBK2 knockdown significantly reduced axon length, while TTBK2 overexpression enhanced elongation. shKIF3A + TTBK2‐OE partially rescued axon length compared with that under shKIF3A alone (*n* = 5 from 3 independent experiments). Scale bars, 10 μm. Data are presented as mean ± SEM. One‐way ANOVA was performed. *p < 0.05, **p < 0.01, ***p < 0.001.

PCs are crucial in neuronal development and are closely linked to axon formation [31, 32]. KIF3A participated in PC assembly (Figure 1A), and *TTBK2* was the regulator in the initial stage of PC formation [4]. To explore whether *TTBK2* promoted the axonal growth of spinal cord neurons through PCs, we extracted spinal cord neurons from C57 fetal mice at 15 days of gestation and generated neuronal cultures. Adenovirus was respectively used to silence KIF3A (shKIF3A) and TTBK2 (shTTBK2), overexpress TTBK2 (TTBK2‐OE), and overexpress TTBK2 on the basis of KIF3A silencing (shKIF3A + TTBK2‐OE). Subsequently, the gene expression in each group was determined using quantitative reverse transcription qRT‐PCR, and the results showed that the adenovirus effectively regulated the expression of KIF3A and TTBK2 (Figure 1B,C).

AC3 is a landmark protein of PCs in the cilia [33]. We used MAP2 to label neuronal dendrites and AC3 to label PCs. The PC structures of the shTTBK2 and shKIF3A groups were destroyed, and the cilia were markedly shortened. The cilia in the TTBK2‐OE group were longer than those in the control group. In contrast, the cilia of cells overexpressing TTBK2 in the presence of shKIF3A (shKIF3A + TTBK2‐OE) were longer than those of shTTBK2 and shKIF3A but shorter than those of the control group (Figure 1D,E). These results suggest that TTBK2 is important in the regulation of PC formation.

Considering the role of PCs in axon formation, we used TUJ1 and MAP2 to label neurons and dendrites, respectively, to observe the effects of TTBK2 on axon growth which not labeled with MAP2. TTBK2 overexpression somewhat promoted axonal growth. However, inhibiting TTBK2 expression significantly reduced the axon length (Figure 1F,G). When the PCs were damaged, TTBK2 overexpression partially abrogated the shortening of the axon (shKIF3A + TTBK2‐OE), suggesting that TTBK2 affects the development of neuronal axons by regulating the PC structure.

### 3.2 TTBK2 Regulates Axonal Development via SHH Signaling Through PCs

**FIGURE 2:**
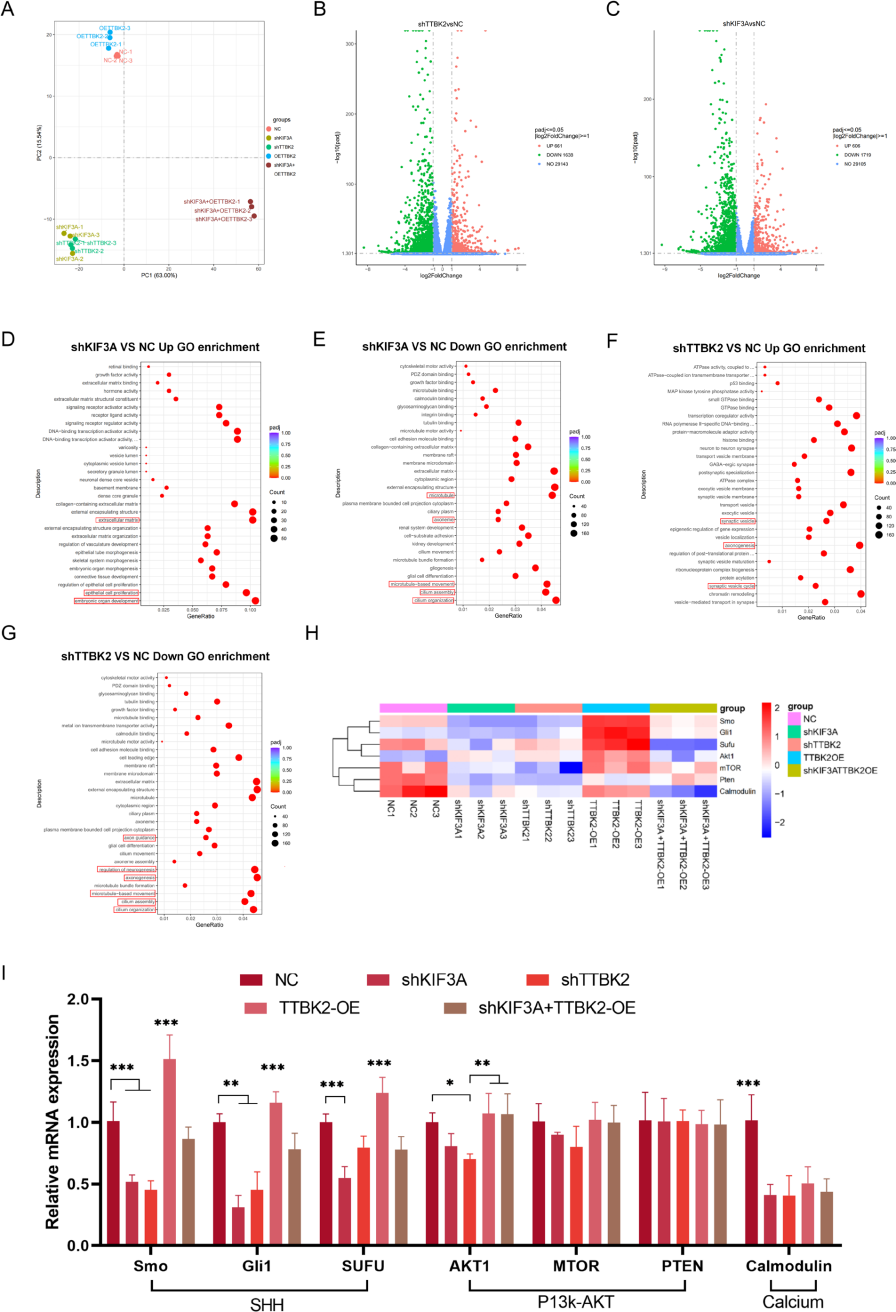
Transcriptomic analysis reveals that TTBK2 regulates axon regeneration via primary cilia–dependent SHH signaling. (A) Principal component analysis (PCA) of transcriptomic profiles in spinal neurons across five experimental groups, showing distinct clustering (*n* = 3 from 3 independent experiments). (B, C) Volcano plots displaying differentially expressed genes (DEGs) between shKIF3A vs. NC and shTTBK2 vs. NC. Significant DEGs were defined as padj ≤ 0.05 and |log_2_ fold change| ≥ 1. (D–G) GO enrichment analysis showing that KIF3A or TTBK2 knockdown significantly downregulated the biological processes associated with cilium assembly, microtubule organization, and axon formation. TTBK2 silencing also suppressed pathways related to axon guidance and neurogenesis. (H) Heat map of key genes in the PI3K‐Akt and Hedgehog pathways. (I) qRT‐PCR validation of key genes in the PI3K‐Akt, Hedgehog, and calcium signaling pathways. SHH‐related genes were more closely correlated with TTBK2 expression (*n* = 3 from 3 independent experiments). Data presentation: Mean ± SEM. One‐way ANOVA was performed. *p < 0.05, **p < 0.01, ***p < 0.001. See also Figures S1–S3.

To explore how TTBK2 regulates the growth of axons through PCs, we sequenced the five groups of spinal cord neurons using RNA‐sequencing (RNA‐seq) and uploaded the sequencing data to the Gene Expression Omnibus database (GEO): GSE301749. Principal component analysis of the gene expression values of all samples revealed that the experimental groups showed evident clustering characteristics at the transcriptional level, indicating that different treatments had significant effects on the transcription group (Figure 2A).

In the differential expression analysis, compared with those of the negative control group (NC), the shKIF3A group had 661 and 1638 upregulated and downregulated genes, respectively. There were 606 upregulated genes and 1719 downregulated genes in the shTTBK2 group (the screening criteria were *p*adj ≤ 0.05, |log2FoldChange| ≥ 1, Figure 2B,C), The results of the comparison between the remaining groups and NC, as well as the results of the clustering heat map, are shown in (Figure S1A–D). To further clarify the function of differential genes, we performed Gene Ontology (GO) enrichment analysis on the differentially expressed genes (DEGs). When *KIF3A* expression was silenced, functions related to extracellular matrix reconstruction, epithelial cell proliferation, and embryonic development were significantly upregulated, whereas those closely related to neuromorphological development, such as cilium assembly, microtubule construction, and axon formation, were significantly downregulated (Figure 2D,E). In contrast, *TTBK2*‐silencing induced the downregulation of cilium assembly, microtubule, and axon formation, significantly inhibited axon guidance and neuron generation, and simultaneously enhanced the release and transport function of synaptic vesicles (Figure 2F,G). Overexpression of TTBK2 demonstrated enhanced functions related to G protein coupling (Figure S1E,F). These results are consistent with the aforementioned morphological observations.

Furthermore, we conducted Kyoto Encyclopedia of Genes and Genome (KEGG) pathway enrichment analysis to determine the signaling mechanism by which TTBK2 mediates PCs to regulate axonal growth. *TTBK2* and *KIF3A* were closely related to PI3K‐Akt and Hedgehog (Figure S2A–F). To verify the changes in pathway activity, we have mapped the heat maps of the key target genes of the relevant pathways (Figure 2H) and used qRT‐PCR to determine the expression levels of key target genes in the pathways identified. The expression of *SHH*‐related genes in the Hedgehog pathway had the most significant change and was highly correlated with *TTBK2* expression (Figure 2I), suggesting that *TTBK2* regulates the axonal development of neurons through the PC‐SHH signaling axis (Figure S3A,B).

**FIGURE 3:**
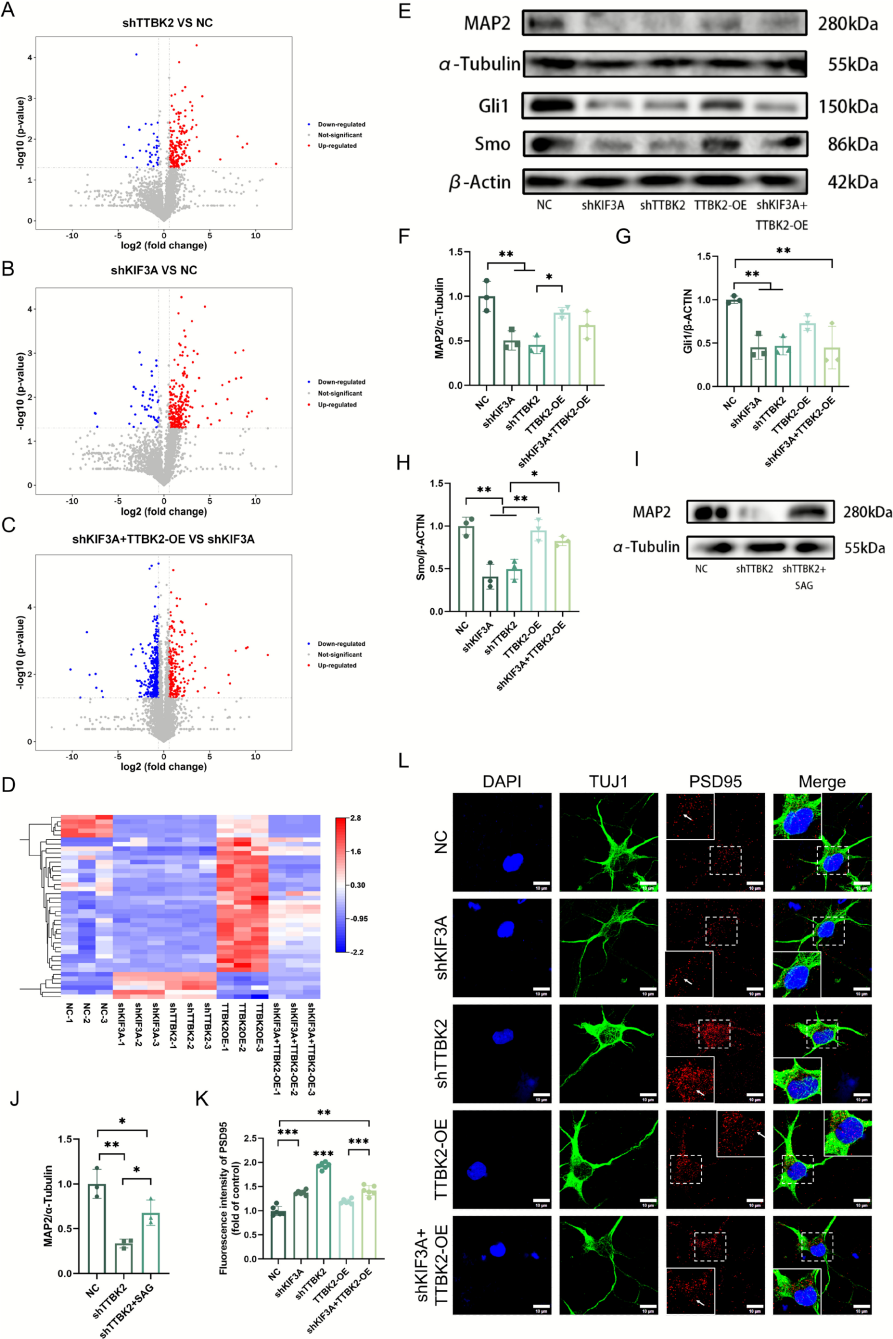
TTBK2 regulates axonal regeneration via the primary cilium–SHH pathway. (A–C) Volcano plots showing differentially expressed proteins between shTTBK2 vs. NC, shKIF3A vs. NC, and shKIF3A + TTBK2‐OE vs. shKIF3A. The *x*‐axis represents log2 (fold change), and the *y*‐axis represents −log10(*p*‐value). Gray dots indicate proteins that did not meet significance thresholds (*p* > 0.05). Blue and red dots indicate downregulated and upregulated proteins, respectively. (D) The heatmap displays differentially expressed proteins identified in each of the four experimental groups relative to the NC control group, with color intensity representing expression levels. (E–H) Western blotting analysis demonstrating significant reductions in MAP2, Gli1, and Smo protein expression in the shTTBK2 group (*n* = 3 from 3 independent experiments). (I, J) Treatment with the SHH pathway agonist SAG restored MAP2 expression in shTTBK2 neurons (*n* = 3, from 3 independent experiments *p < 0.05). (K, L) Representative immunofluorescence images of spinal neurons stained for TUJ1 (green), PSD95 (red), and DAPI (blue). TTBK2‐OE group showed markedly increased PSD95 expression compared to other groups (*n* = 6 from 3 independent experiments). Scale bar, 5 μm. Data are presented as mean ± SEM. One‐way ANOVA was used for statistical analysis. *p < 0.05, **p < 0.01, ***p < 0.001.

To verify the above mechanism at the protein level, we conducted a quantitative protein omics analysis on five groups of spinal cord neuron samples. The distribution and functional orientation of differentially expressed proteins (DEPs) were demonstrated using volcano plots. Using established screening criteria (fold change ≥ 1.5 or ≤ 0.67, *p* ≤ 0.05), the shTTBK2 group exhibited 184 and 38 upregulated and downregulated proteins, respectively, compared with those of the NC group (Figure 3A). There were 232 upregulated and 38 downregulated proteins in the shKIF3A group, and the changes in DEPs were statistically significant (Figure 3B). Compared with shKIF3A, there were 195 upregulated proteins and 357 down‐regulated proteins in the shKIF3A‐TTBK2‐OE group (Figure 3C). Screening for common differentially expressed proteins between the experimental and NC groups was followed by the generation of a corresponding heatmap (Figure 3D).

We evaluated the expression of proteins of the SHH signaling pathway to verify the functional correlation of the protein results. The expression levels of the key SHH signaling molecules, Smo, Gli1, and detected the expression of MAP2, which is related to axon regeneration [34], were significantly downregulated (Figure 3E–H) in the shKIF3A and shTTBK2 groups. Furthermore, to explore whether the inhibition of axonal development caused by TTBK2 silencing was reversible, we applied the SHH pathway agonist, smoothened agonist (SAG), activates the Shh pathway by relieving Patched1 (Ptch1)–mediated inhibition of Smo, to the shTTBK2 group of cells. SAG partially restored the expression of MAP2 protein (Figure 3I,J), suggesting that the SHH pathway plays a key role in TTBK2‐mediated axonal regulation.

Furthermore, the five groups of neuronal cultures were immunostained for PSD95 to evaluate the expression status of this synapse‐related protein. The expression levels of PSD95 in the shKIF3A and shTTBK2 groups were significantly increased (Figure 3K,L), suggesting that functional damage to PCs enhances the synaptic excitability of neurons, consistent with the literature [35]. Although the expression of PSD95 in the TTBK2‐OE group was significantly lower than that in the shTTBK2 group, it showed no significant differences compared with that in the NC group. Based on these results, we believe that TTBK2 may affect MAP2 expression and microtubule homeostasis by regulating the PC‐dependent SHH signaling pathway, thus modulating the growth and development of neuronal axons.

### 3.3 TTBK2^fl^ ^/fl^‐Cre ERT ^+/−^mouse Model of Spinal Cord Lateral Hemisection

**FIGURE 4:**
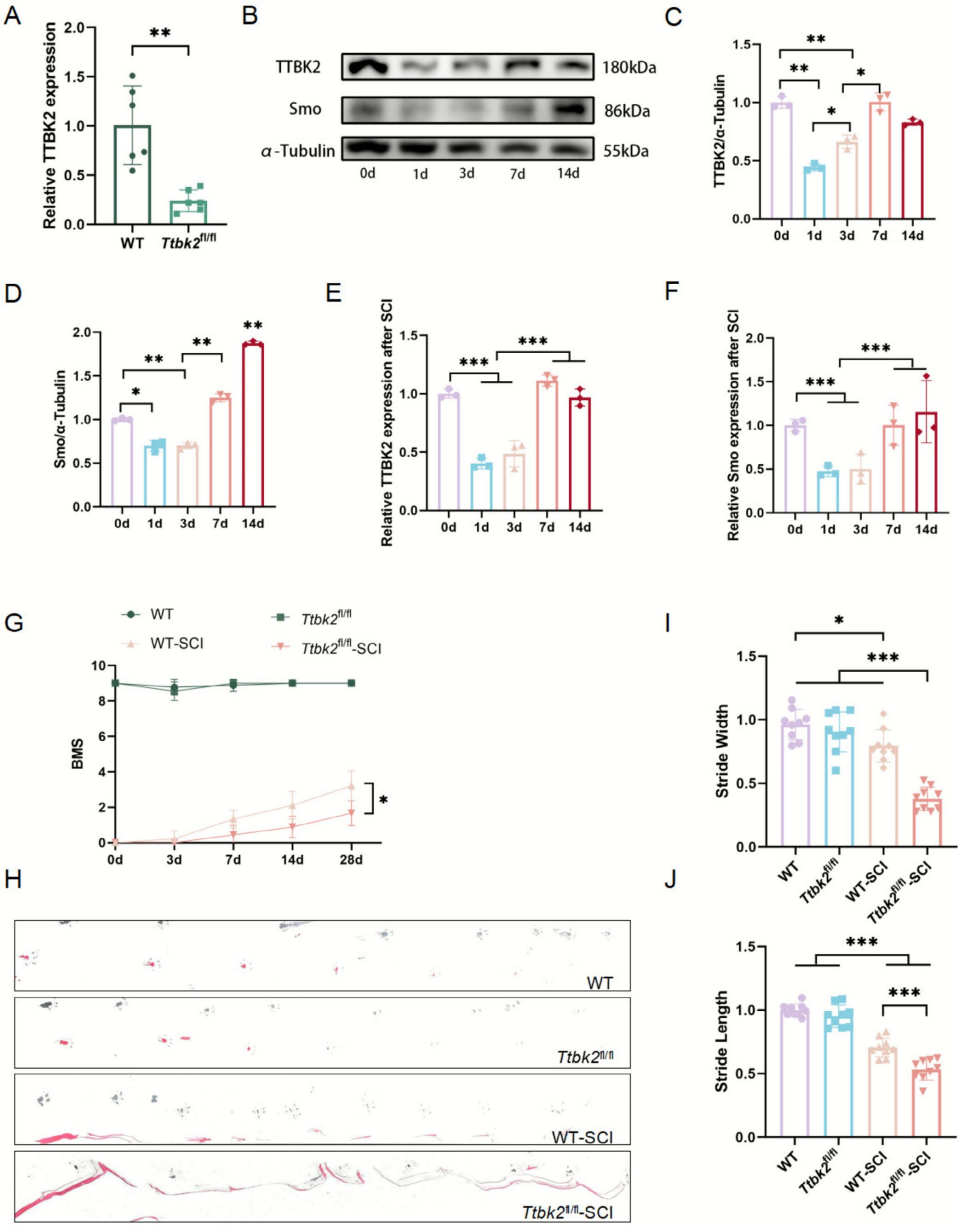
Generation and behavioral analysis of the *Ttbk2* ^fl/fl^‐Cre ERT^+/−^ spinal cord hemisection model. (A) qRT‐PCR analysis confirming TTBK2 gene knockout in *Ttbk2* ^fl/fl^‐Cre ERT^+/−^ mice following tamoxifen injection (*n* = 6 from six mice in each group). (B–D) Western blotting revealed a biphasic expression pattern of TTBK2 and Smo after SCI, characterized by an initial decrease followed by a subsequent increase (*n* = 3 from 3 independent experiments). (E, F) The qRT‐PCR results of the injury site showed that the expressions of TTBK2 and Smo decreased first and then increased after SCI (*n* = 3, from 3 independent experiments). (G) BMS revealed significantly better locomotor recovery in WT‐SCI mice compared to *Ttbk2* ^fl/fl^‐SCI mice at 28 days post‐injury (*n* = 9 from nine mice in each group). Two‐way ANOVA was used for statistical analysis. (H) Gait analysis showed that while WT‐SCI mice retained partial right hindlimb dragging, their stride patterns resembled normal mice. In contrast, *Ttbk2* ^fl/fl^‐SCI mice exhibited continuous dragging with indistinct footprints. (I, J) Quantification of stride length and step width demonstrated significantly reduced locomotor parameters in *Ttbk2* ^fl/fl^‐SCI mice compared to WT‐SCI controls (*n* = 9 from nine mice in each group). Bars and errors represent mean ± SEM. *p < 0.05, **p < 0.01, ***p < 0.001 (one‐way ANOVA).

PCs are key regulatory factors in the early development of brain neurons and represent the core organelles for cell cycle regulation [36]. However, whether PCs play a role in endogenous repair after SCI is unclear. To explore the role of PCs in SCI, we used gene‐targeting technology and hybridized with Rosa‐CreERT2^+/+^ mice, resulting in *Ttbk2* ^fl/fl^‐Rosa‐CreERT2^+/−^ conditional knockout experimental mice. Genotyping (Figure S4A) was performed on the 14th day after birth. Then we established a mouse model of *Ttbk2* ^fl/fl^‐Rosa‐CreERT2^+/−^ 10th thoracic vertebrae (T10) spinal cord hemi‐section injury (Figure S4B). At 4 weeks of age, *Ttbk2* conditional knockout was induced through tamoxifen injection, and the knockout efficiency was verified via qRT‐PCR and Western blot. The expression of the *Ttbk2* gene in the spinal cord of injected mice was significantly downregulated (Figures 4A and S4C).

To characterize the temporal dynamics of TTBK2 and Shh signaling after SCI, we quantified the expression of TTBK2 and the key downstream effector of Shh signaling—Smo at 0, 1, 3, 7, and 14 days following spinal cord hemisection using Western blot and qRT‐PCR analyses. Western blot results showed that both TTBK2 and Smo were markedly reduced at 1 and 3 days post‐injury, partially recovered by Day 7 without reaching pre‐injury levels, and exhibited a significant increase only for Smo at Day 14 (Figure 4B–D). The temporal pattern was further validated at the transcriptional level. qRT‐PCR analysis revealed a comparable pattern, with transcript levels declining during the acute phase and gradually returning toward baseline in the subacute phase, though no significant differences from pre‐injury levels were detected at Day 14 (Figure 4E,F). The concordant protein and mRNA changes indicate a coordinated regulation of TTBK2 and SHH‐pathway activity following SCI.

To evaluate the function of TTBK2 in SCI repair, we established a T10 spinal cord injury model (Figure S4D,E) and assigned the mice to four groups: *Ttbk2* ^fl/fl^‐Rosa‐CreERT2^−/−^ (wild type, WT), *Ttbk2* ^fl/fl^‐Rosa‐CreERT2^−/−^ + SCI (WT‐SCI), *Ttbk2* ^fl/fl^‐Rosa‐CreERT2^+/−^ (*Ttbk2* ^fl/fl^), and *Ttbk2* ^fl/fl^‐Rosa‐CreERT2^+/−^ + SCI (*Ttbk2* ^fl/fl^‐SCI). At 14 days after SCI gross examination revealed no obvious differences in the macroscopic appearance of spinal cord tissues between the WT‐SCI and *Ttbk2* ^fl/fl^‐SCI groups (Figure S4E). The BMS score was used to evaluate the recovery of motor function in the right hind limb [37]. On the 28th day, the *Ttbk2* ^fl/fl^‐SCI and WT‐SCI groups had BMS scores of 1–2 and 2–4 points, respectively, showing a statistically significant difference (*p* < 0.05, Figure 4G).

Furthermore, the red ink footprint analysis was used to evaluate the gait of mice. We painted red ink on the right hind limb of the mice, made them walk on the runway, and measured their step size and width (Figure S4F). The results showed that on the 14th day, the gait patterns of mice in all SCI groups were disordered and could not be restored to normal. Mice in the WT‐SCI group had fewer drag marks, and the toe spacing was similar to that in the sham operation group. However, the *Ttbk2* ^fl/fl^‐SCI group showed continuous dragging with widened toe spacing (Figure 4H–J), suggesting that *TTBK2* deficiency hindered functional recovery after SCI, consistent with the BMS score. In addition, *Ttbk2* ^fl/fl^‐SCI mice showed apparent hind limb spasm symptoms (Figure S4G), which may be related to enhanced neuronal excitability caused by excessive activation of synaptic signals [38].

Notably, 28 days after tamoxifen induction, *Ttbk2* ^fl/fl^ mice showed evident weight gain. From these mice, we extracted and stained the heart, liver, and kidney with hematoxylin and eosin (HE). We observed lipid droplets in cells in the organs (Figure S5A,B), suggesting that the PC dysfunction caused a disorder in the GPCR‐related signaling pathway, leading to excessive fat accumulation in mice [39, 40].

### 3.4 Ttbk2 Conditional Knockout Inhibits Axonal Regeneration and Induces Postsynaptic Density Protein Expression

**FIGURE 5:**
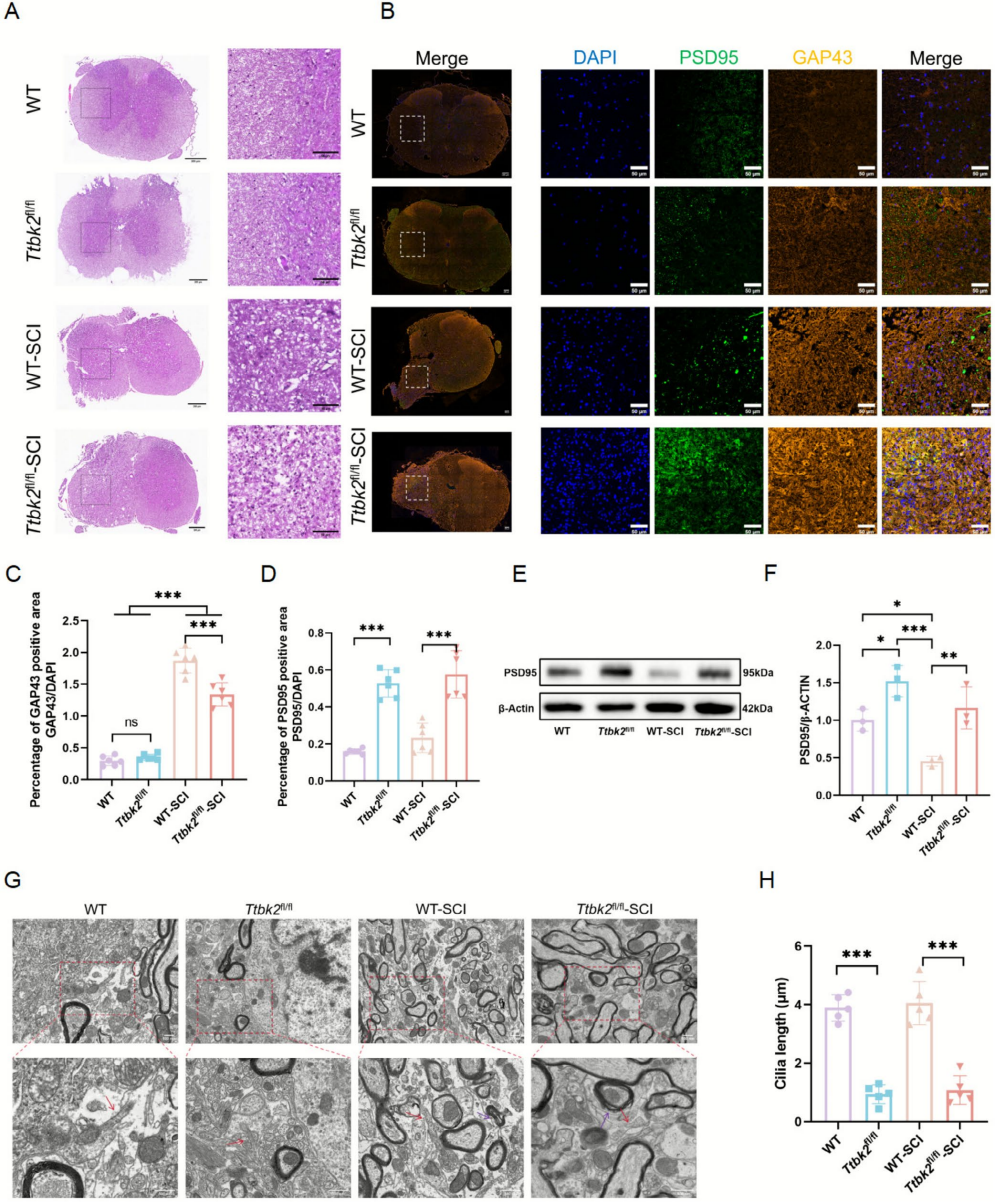
*Ttbk2* conditional knockout impairs axonal regeneration and enhances synaptic excitability after SCI. (A) Representative H&E staining of spinal cord cross‐sections at the injury site (*n* = 3 from three mice in each group). Scale bars: Left, 200 μm; right, 50 μm. (B) Representative immunofluorescence images of frozen T10 spinal cord sections stained for GAP43 (orange), PSD95 (green), and DAPI (blue) (*n* = 6 from six mice in each group). Scale bars: Left, 200 μm; right, 50 μm. (C, D) Semi‐quantitative analysis showed that GAP43 expression was elevated in injured groups, particularly in WT‐SCI. PSD95 expression was higher in *Ttbk2* ^fl/fl^ and *Ttbk2* ^fl/fl^‐SCI mice than in the WT and WT‐SCI groups (*n* = 6 from six mice in each group). (E, F) Western blotting confirmed elevated PSD95 in *Ttbk2* knockout mice. Interestingly, PSD95 levels in WT‐SCI mice were lower than those in WT controls, differing from immunofluorescence results (*n* = 3 from three mice in each group). (G) TEM revealed incomplete primary cilia in *Ttbk2* ^fl/fl^ and *Ttbk2* ^fl/fl^‐SCI neurons (red arrows). WT‐SCI and *Ttbk2* ^fl/fl^‐SCI groups also exhibited disrupted neuronal membranes, spindle‐shaped myelin, and loosened myelin layers. Scale bars: 500 nm. (H) Statistical analysis of ciliary length in TEM experiment. Bars and errors represent mean ± SEM. *p < 0.05, **p < 0.01, ***p < 0.001 (one‐way ANOVA).

To evaluate the effect of *Ttbk2* knockout on the tissue structure after SCI, we perfused the mice in each group on the 14th day after injury and performed histology with HE to observe changes in the spinal cord. Compared to the sham operation group, the continuity of spinal gray matter in the WT‐SCI and *Ttbk2* ^fl/fl^‐SCI groups was destroyed, and vacuolar lesions of different sizes appeared in the white matter (Figure 5A).

To evaluate the regenerative ability of neurons and synaptic structure changes, we performed GAP43 and PSD95 immunostaining on the injured T10 segment in mice. GAP43 expression was significantly greater in the WT‐SCI group than that in the *Ttbk2* ^fl/fl^‐SCI group, whereas PSD95 was highly expressed in both groups, particularly in the *Ttbk2* ^fl/fl^‐SCI group (Figure 5B–D). Western blotting confirmed that the protein levels of PSD95 in the *Ttbk2* ^fl/fl^‐SCI group were significantly higher than those in the WT‐SCI group (Figure 5E,F). These results suggest that *Ttbk2* knockout significantly inhibits axonal regeneration and increases expression of synaptic excitatory proteins.

Transmission electron microscopy (TEM) revealed ultrastructural changes in the spinal cord of mice. Destroyed PCs were found in the nerve cells of the *Ttbk2* ^fl/fl^ and *Ttbk2* ^fl/fl^‐SCI groups; the length of cilia was significantly lower than that of the WT group, *p* < 0.001. In the WT‐SCI and *Ttbk2* ^fl/fl^‐SCI groups, the integrity of the neuronal membranes was damaged, the local myelin showed spindle‐shaped changes, the thickness of myelin was relatively uniform, there was slight structural looseness, and some axons in the myelin were separated from their membrane structures accompanied by slight axonal atrophy (Figure 5G,H). Luxol Fast Blue (LFB) staining showed that the *Ttbk2* ^fl/fl^ group exhibited more demyelinating changes than did the WT group (Figure S6).

### 3.5 TTBK2‐SHH‐MAP2 Axis Regulates Endogenous Repair After SCI

**FIGURE 6:**
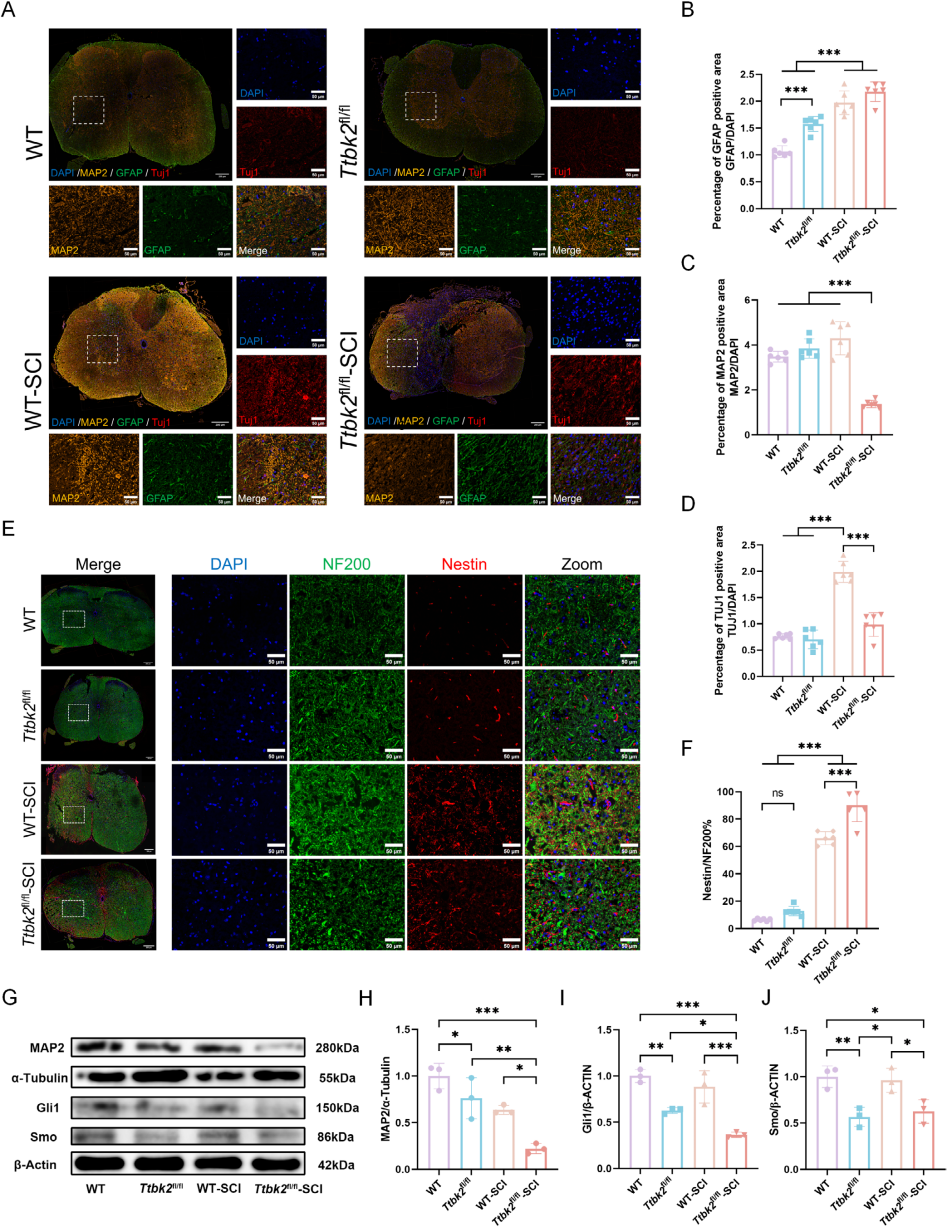
The TTBK2–SHH–MAP2 axis regulates endogenous neuronal repair following SCI. (A) Representative immunofluorescence images of frozen spinal cord sections stained with GFAP (green), MAP2 (orange), TUJ1 (red), and DAPI (blue). Scale bars: Left, 200 μm; right, 50 μm (*n* = 6 from six mice in each group). (B–D) The quantification of immunostaining showed increased GFAP in all injured groups. MAP2 levels in WT‐SCI were comparable to those in uninjured controls, while *Ttbk2* ^fl/fl^‐SCI showed a marked reduction. TUJ1 staining indicated significantly higher immature neuron proportion in WT‐SCI than in other groups (*n* = 6 from six mice in each group). (E, F) Co‐staining of NF200 (green) and Nestin (red) revealed elevated neural progenitor marker Nestin in injured groups. *Ttbk2* ^fl/fl^‐SCI mice exhibited a higher Nestin/NF200 ratio than did WT‐SCI mice (*n* = 6 from six mice in each group). (G–J) Western blotting results confirmed that MAP2, Smo, and Gli1 protein levels were significantly reduced in *Ttbk2* ^fl/fl^‐SCI mice, indicating SHH pathway suppression (*n* = 3 from three mice in each group). Bars and errors represent mean ± SEM. *p < 0.05, **p < 0.01, ***p < 0.001 (one‐way ANOVA).

The endogenous repair ability after SCI largely depends on the activation of astrocytes in the injured area and the activation, migration, and differentiation of endogenous neural stem cells [41]. As a microtubule‐associated protein, MAP2 is closely related to the stability of neuronal microtubules and axon formation [42, 43]. Therefore, we used immunofluorescence staining of GFAP (astrocytes), TUJ1 (neurons), and MAP2 (neuronal dendrites) to evaluate neuronal regeneration in the injured area. GFAP expression in the WT‐SCI and *Ttbk2* ^fl/fl^‐SCI groups was significantly increased compared with that in the WT group, suggesting that the reactive activation of astrocytes was enhanced, which was closely related to scar formation after injury. Although MAP2 had similar expression levels in the WT‐SCI and sham operation groups, the expression was significantly decreased in the *Ttbk2* ^fl/fl^‐SCI group. Similarly, in the *Ttbk2* ^fl/fl^‐SCI group, the gray matter structure in the injured area was absent, and there was almost no MAP2 signal in some areas. TUJ1, labeling newborn neurons, was significantly more abundant in the WT‐SCI group than in the *Ttbk2* ^fl/fl^‐SCI group, suggesting that *Ttbk2* deletion inhibited the generation of newborn neurons (Figure 6A–D).

Furthermore, we used NF200 (a mature neuron marker) and nestin (a neural stem cell marker) to evaluate the differentiation potential of endogenous neural stem cells. The number of nestin‐positive cells in the *Ttbk2* ^fl/fl^‐SCI group was significantly higher than that in the WT‐SCI group (Figure 6E,F), suggesting that *Ttbk2* knockout may hinder the transformation of neural stem cells into neurons. This may be related to the destruction of PCs, leading to the suppression of SHH signaling and affecting the differentiation of neural stem cells into neurons.

To investigate whether *Ttbk2* regulates the SHH signaling pathway through PCs in vivo, we measured the expression levels of Smo and Gli1 proteins in each group of mice 14 days after the injury and detected the expression of MAP2, which is related to axon regeneration [34, 44]. MAP2 protein expression was significantly decreased in the *Ttbk2* ^fl/fl^‐SCI group, and the expression of Smo and Gli1 in the *Ttbk2* ^fl/fl^ and *Ttbk2* ^fl/fl^‐SCI groups was also significantly decreased, confirming that *Ttbk2* regulates the expression of neuronal structure‐related proteins through the PC‐mediated SHH pathway (Figure 6G–J).

### 3.6 Ttbk2 Regulates Axon Guidance and Spinal Circuit Remodeling via PCs

**FIGURE 7:**
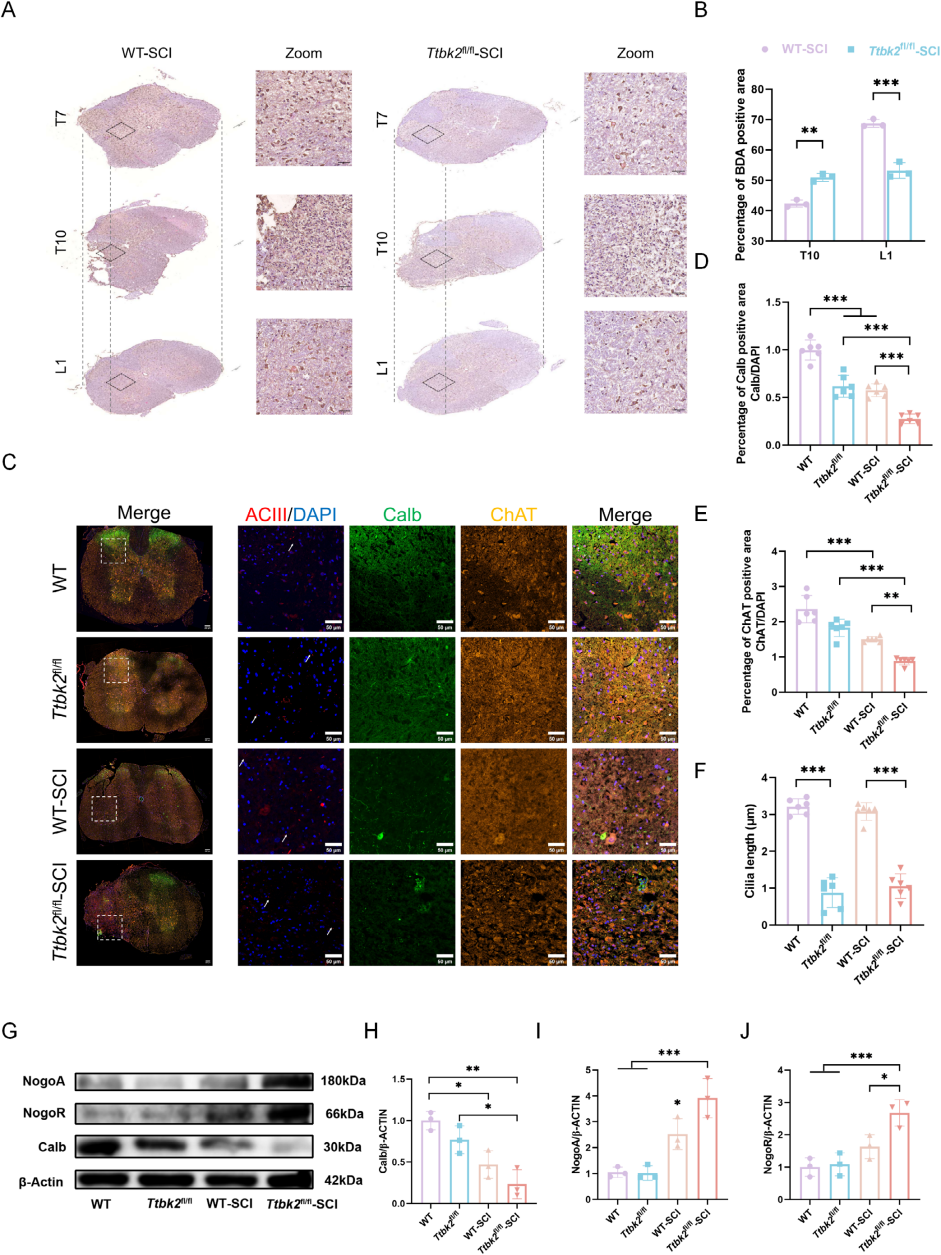
TTBK2 modulates axon guidance and spinal circuit remodeling via primary cilia. (A, B) BDA tracing showed higher axonal labeling in *Ttbk2* ^fl/fl^‐SCI mice at the T10 level but fewer labeled axons at L1 compared with those in WT‐SCI, suggesting impaired long‐distance axonal projection. Scale bars: Left, 200 μm; right, 50 μm (*n* = 3 from three mice in each group). (C) Representative images showing co‐staining of Calbindin (green, interneurons), ACIII (red, PCs), ChAT (orange, motor neurons), and DAPI (blue). White arrows denote primary cilia. Scale bars: Left, 200 μm; right, 50 μm (*n* = 6 from six mice in each group). (D–F) Quantification showed decreased Calbindin and ChAT in WT‐SCI mice compared with those in WT mice. While Calbindin expression did not differ significantly between WT‐SCI and *Ttbk2* ^fl/fl^‐SCI, ChAT levels were lower in the latter, and PC length of *Ttbk2* knockout mice was significantly lower than that of wild type mice (*n* = 6 from six mice in each group). (G–J) Western blotting analysis confirmed reduced Calbindin and increased expression of axon growth inhibitors NogoA and NogoR in *Ttbk2* ^fl/fl^‐SCI (*n* = 3 from three mice in each group). Bars and errors represent mean ± SEM. *p < 0.05, **p < 0.01, ***p < 0.001 (one‐way ANOVA).

After SCI, the migration and orientation of axons play key roles in the reconstruction of neural circuits. To explore whether *Ttbk2* affects the remodeling of the spinal nerve loop through PCs, we used a biotinylated dextran amine (BDA) tracer technique to analyze the distribution of positive neurons in the injured segment (T10) and the lower L1 segment to assess axon extension and the reconstruction of the nerve conduction. Compared with those in the WT‐SCI group, the number of BDA‐positive neurons in the *Ttbk2* ^fl/fl^‐SCI group was significantly increased in the T10 segment but was significantly decreased in the L1 segment (Figure 7A,B), suggesting that axons failed to form effective downstream connections after *Ttbk2* deletion and may form abnormal lateral projections instead.

Confocal microscopy further revealed that the expression of the PC marker protein AC3 was nearly absent in the *Ttbk2* ^fl/fl^ and *Ttbk2* ^fl/fl^‐SCI groups, suggesting that the PC structure was critically damaged. Similarly, the expression levels of interneurons labeled for Calb and spinal motor neurons labeled for Chat in the *Ttbk2* ^fl/fl^‐SCI group were significantly decreased (Figure 7C–F). Western blot analysis confirmed these observations. The levels of Calb protein in the *Ttbk2* ^fl/fl^‐SCI group of mice were significantly decreased, while those of NogoA and its receptor, NogoR, were significantly increased (Figure 7G–J). Collectively, these results indicate that *Ttbk2* deletion interferes with neuronal subtype differentiation and axonal orientation by destroying the PC structure, and affects neural circuit reconstruction after SCI.

### 3.7 Pharmacological Inhibition or Activation of the SHH Pathway Modulates MAP2 Expression After SCI

To further determine whether SHH signaling is required and/or sufficient for neuronal integrity after SCI, we examined MAP2 expression across five experimental groups (WT, WT‐SCI, WT‐SCI + Cyclopamine, *Ttbk2* ^fl/fl^‐SCI, *Ttbk2* ^fl/fl^‐SCI + SAG). Western blotting analysis revealed a graded pattern of MAP2 levels (Figure S7A). WT animals showed the highest MAP2 expression. WT‐SCI exhibited a moderate reduction. WT‐SCI + Cyclopamine and *Ttbk2* ^fl/fl^‐SCI displayed the lowest MAP2 levels, both significantly reduced compared with WT‐SCI (*p* < 0.05). Importantly, SAG treatment partially rescued MAP2 expression in *Ttbk2* ^fl/fl^‐SCI mice, resulting in significantly higher MAP2 levels compared with untreated *Ttbk2* ^fl/fl^‐SCI animals (*p* < 0.05), although still lower than WT‐SCI. This graded response indicates that SHH signaling is necessary for maintaining neuronal structural integrity after SCI (Cyclopamine decreases MAP2), and SHH activation is partially sufficient to rescue MAP2 loss in the context of *Ttbk2* deficiency, placing SHH downstream of TTBK2‐dependent ciliary regulation (Figure S7B).

### 3.8 Neuronal Ttbk2 Depletion Recapitulates the Functional and Anatomical Deficits Observed in Global Knockout Mice

To determine whether neuronal loss of *Ttbk2* is sufficient to account for the impaired recovery after SCI, we generated a focal, neuron‐specific conditional knockout by delivering AAV9‐hSyn‐Cre into *Ttbk2* ^fl/fl^ mice prior to injury (AAV‐Cre‐SCI; Figure S8A–C). Behavioral and anatomical outcomes were compared with AAV9‐hSyn–injected controls (NC‐SCI) and with *Ttbk2* ^fl/fl^‐SCI mice.

BMS assessments showed that both AAV‐Cre‐SCI and *Ttbk2* ^fl/fl^‐SCI mice displayed significantly reduced locomotor scores relative to NC‐SCI mice across the entire recovery period (*p* < 0.01). Notably, no significant differences were detected between AAV‐Cre‐SCI and *Ttbk2* ^fl/fl^‐SCI groups (*p* < 0.05), indicating that neuronal deletion of *Ttbk2* is sufficient to phenocopy the behavioral deficits observed in global knockout mice (Figure S8D).

BDA tracing further revealed comparable disruptions in corticospinal tract projections between the neuron‐specific and global knockout groups. At the T10 level, both AAV‐Cre‐SCI and*Ttbk2* ^fl/fl^‐SCI mice exhibited increased BDA‐positive fiber density relative to NC‐SCI controls, whereas at the L1 level, both groups showed markedly reduced caudal BDA labeling (*p* < 0.01). Consistent with the behavioral data, no significant differences were observed between the two knockout groups at any spinal level (Figure S9A,B).

Western blot analysis across six groups (NC, NC‐SCI, AAV‐Cre, AAV‐Cre‐SCI, *Ttbk2* ^fl/fl^, *Ttbk2* ^fl/fl^‐SCI) showed that MAP2 expression was significantly reduced in both AAV‐Cre‐SCI and *Ttbk2* ^fl/fl^‐SCI mice relative to NC‐SCI mice (*p* < 0.05), with no detectable differences between the two *Ttbk2*‐deficient SCI groups (*p* < 0.05). These molecular findings further support the conclusion that neuronal loss of *Ttbk2* is sufficient to compromise neuronal integrity after SCI (Figure S9C,D).

Collectively, these results demonstrate that neuron‐specific *Ttbk2* depletion reproduces the major behavioral, anatomical, and molecular phenotypes observed in global knockout mice, indicating that *Ttbk2* functions primarily within neurons to support recovery and neural circuit integrity following SCI.

## 4 Discussion

This study systematically revealed the critical role of TTBK2 in regulating axonal regeneration, synaptic excitability, and neural circuit reorganization following SCI through its modulation of PCs and the SHH pathway. It further demonstrated that PCs are essential in neurodevelopment and play a pivotal regulatory role in SCI neural repair and regeneration. For the first time, through a series of in vitro and in vivo experiments, we demonstrated that TTBK2 deficiency leads to the disruption of the PC structure, resulting in the downregulation of SHH signaling, impaired axonal regeneration, aberrant increased expression of synaptic excitatory proteins, and the inhibition of neural circuit rewiring, ultimately leading to failed recovery of motor function after injury. This represents the first report internationally on the correlation between TTBK2, PC, and neural repair following SCI.

Previous studies have well established *TTBK2* as a key initiator of ciliogenesis and a critical gene in the maintenance of PC homeostasis [20, 22]. As the only non‐motile ciliary structure, PCs play significant roles in various stages of CNS development. Early in the embryonic stage, PCs are central in regulating the differentiation, migration, and polarity establishment of neural precursor cells [45, 46]. During cortical development, PCs act as “sensing antennas” for pathways such as SHH and Wnt cascades, integrating external morphogenic signals to precisely regulate the fates of neural stem cells [13, 47, 48]. Moreover, the cilia exhibit the ability to modulate neuronal movement in the mature cortex [49]. Studies indicate that the PCs of hippocampal pyramidal neurons connect with serotonergic axons, participate in synaptic transmission [50], and functionally couple with glutamate receptor signaling [51]. Furthermore, cilium‐defective mouse models exhibit abnormal synaptic vesicle transport and enhanced excitatory synapses, suggesting that the cilia function during neurodevelopment and in the maintenance of functional homeostasis in mature neural networks [35, 52]. These studies have clarified the crucial regulatory role of PCs in CNS development; however, their function and significance in CNS injuries, particularly SCI, remain unexplored. The present study aimed to address these literature gaps.

Axonal regeneration and elongation are key determinants of successful repair after SCI. In this study, we investigated the upstream and downstream pathways associated with PCs in axonal growth. We first silenced and overexpressed TTBK2 in vitro using shTTBK2 and TTBK2‐OE, respectively, and silenced PCs using shKIF3A. These resulted in significantly shortened axonal length in the shTTBK2 and shKIF3A groups and increased axonal length in the TTBK2‐OE group, demonstrating that TTBK2‐mediated PCs explicitly promote axonal growth. As hubs for activating multiple cellular pathways, PCs influence behaviors such as cell proliferation, migration, or axonal growth by regulating different signaling cascades. To investigate the neuronal pathways activated by TTBK2/PC, we used transcriptomic and proteomic analyses for screening and validation. Transcriptomic GO analysis indicated that TTBK2/PC reduces axonal growth, while KEGG analysis revealed that TTBK2 knockdown and PC disruption commonly downregulate pathways including SHH (Hedgehog), PI3K‐AKT, and calcium signaling. Protein–protein interaction network analysis displayed the SHH pathway as the most relevant to axonal growth in the TTBK2/PC context. Subsequent qPCR validation confirmed that TTBK2 and PC knockdown significantly downregulated SHH downstream components Gli1 and Smo, while TTBK2 overexpression showed no difference compared with the events in the control group. These transcriptomic screening and verification further illustrate the regulatory role of the TTBK2/PC/SHH pathway on neuronal cell behavior. The proteomic results indicated that TTBK2/PC knockdown caused the significant downregulation of the protein MAP2. The detection of MAP2 expression following the activation and inhibition of the SHH pathway showed that shTTBK2 and shKIF3A inhibited MAP2 expression, while the SHH pathway agonist SAG rescued the shTTBK2‐induced decrease in MAP2 expression, indicating that the TTBK2/PC/SHH axis is a vital cellular pathway promoting neuronal axonal growth.

Furthermore, the transcriptomic screening revealed a unique phenomenon: GO analysis indicated that TTBK2 knockdown and PC disruption significantly enhanced neuronal synaptic excitability function, suggesting a close relationship between TTBK2‐mediated PCs and synaptic excitability. Therefore, we used immunofluorescence staining to determine the expression of the synaptic protein PSD95 in transfected neurons. The results showed that PSD95 expression was significantly higher in the shTTBK2 and shKIF3A groups compared to the NC group, consistent with the transcriptomic data and indicating that disruption of TTBK2 and PC causes enhanced synaptic excitation. Recent studies have shown the significance of PCs in neuronal excitability and synaptic transmission. Kumamoto et al. demonstrated that PCs participate in the establishment and maintenance of circuit connectivity and excitability in the postnatal brain [51]. Bowie et al. found that PCs in cerebellar Purkinje neurons are necessary for maintaining neuronal excitability in the inferior olivary nucleus of the medulla oblongata [22]. Tereshko et al. showed that disrupting PC signaling in cortical pyramidal neurons rapidly enhanced excitatory glutamatergic input and increased neuronal spontaneous firing, without significantly affecting inhibitory synaptic transmission [35]. These findings confirm the key role of PCs in regulating neuronal excitability, particularly excitatory synapses. Our results further suggest an important link between PCs and synaptic excitability, providing a basis for future research into the regulatory role of PCs in synaptic excitation and signaling during neural development and regeneration.

Our time‐course analysis reveals a dynamic, injury‐phase–dependent regulation of TTBK2 and SHH signaling after SCI. Both TTBK2 and SMO showed a pronounced reduction during the acute phase (1–3 dpi), a period marked by membrane disruption, inflammatory activation, and widespread ciliary disassembly. This coordinated downregulation indicates that early loss of ciliary structure and signaling competence may underlie the diminished intrinsic regenerative capacity characteristic of this stage.

By 7 dpi, expression of both proteins began to recover, reflecting a partial restoration of ciliary architecture and SHH responsiveness as the tissue environment shifts from acute inflammation to subacute repair. Notably, SMO exhibited a more substantial rebound by 14 dpi, suggesting that SHH signaling becomes increasingly engaged during later phases associated with axonal sprouting and circuit reorganization. The consistent trajectories observed at both the protein and mRNA levels support a model in which the TTBK2–cilium–SHH axis undergoes active, phase‐specific modulation rather than persistent suppression after injury.

These findings establish a baseline temporal profile of ciliary and SHH‐pathway dynamics across the critical post‐injury window and provide mechanistic context for the impaired regeneration observed in *Ttbk2*‐deficient mice. The temporal alignment of TTBK2 downregulation, reduced SHH activity, and functional deficits underscores the dependence of neuronal regenerative capacity on intact ciliary homeostasis.

To further explore the regulatory role of TTBK2‐mediated PCs in neural regeneration after SCI, we constructed *Ttbk2* ^fl/fl^ conditional‐knockout mice and established a spinal cord hemisection injury model to investigate motor function and neural repair mechanisms in each group. We found that motor function recovery in the *Ttbk2* ^fl/fl^‐SCI group was significantly lower than in the WT‐SCI group, indicating that TTBK2 and PC are crucial in endogenous neural repair and regeneration after SCI. Endogenous repair mechanisms after SCI primarily include neuronal survival, axonal growth, and circuit reconstruction; thus, we investigated these aspects in the present study. We found that IN and MN expression levels were significantly downregulated in the *Ttbk2* ^fl/fl^‐SCI group. This suggests that TTBK2/PC activates neuroprotective pathways after injury, preserving the number of functional interneurons and motor neurons in the injured area—a prerequisite for initiating endogenous repair. Subsequently, we assessed axonal regeneration using fluorescence and WB detection and found that MAP2 expression was significantly lower in the *Ttbk2* ^fl/fl^‐SCI group than in the WT‐SCI group. This aligns with our in vitro results and further demonstrates that TTBK2/PC promotes axonal regeneration after SCI, forming the foundation for subsequent circuit reconstruction. Furthermore, we validated synaptic excitability in vivo, with the results consistent with those of the in vitro studies; the *Ttbk2* ^fl/fl^ group exhibited enhanced synaptic excitation. Some studies suggest a link between synaptic excitation and spasticity [53], consistent with our study highlighting widespread post‐injury spasticity in *Ttbk2* ^fl/fl^‐SCI mice. We speculate that TTBK2 and PCs may play a regulatory role in post‐SCI spasticity, which is a direction for our subsequent research. Finally, using biotinylated dextran amine (BDA) tracing technology, we validated neural circuit reconstruction after SCI. The results showed that the proportion of BDA‐positive cells caudal to the injury was significantly lower in the *Ttbk2* ^fl/fl^‐SCI group than in the WT‐SCI group, while it was higher at the injury site in the *Ttbk2* ^fl/fl^‐SCI group.

Our pharmacological findings further substantiate the involvement of the SHH pathway in TTBK2‐dependent regulation of neuronal integrity after SCI. In WT‐SCI mice, SHH inhibition with Cyclopamine markedly decreased MAP2 expression, supporting the requirement of SHH signaling for post‐injury neuronal preservation. Conversely, pharmacological activation of SHH signaling with SAG partially restored MAP2 levels in *Ttbk2* ^fl/fl^‐SCI mice, indicating that SHH activation is at least partially sufficient to compensate for the loss of TTBK2. The graded MAP2 responses across the five experimental groups are consistent with a model in which TTBK2 maintains ciliary architecture and thereby modulates downstream SHH signaling essential for neuronal maintenance.

The neuron‐specific loss‐of‐function experiments further clarified the cellular basis of *Ttbk2*‐dependent effects during SCI repair. Targeted delivery of AAV9‐hSyn‐Cre into the spinal cord of *Ttbk2* ^fl/fl^ mice generated a localized neuronal knockout that recapitulated the major deficits observed in global *Ttbk2* deficiency—including impaired locomotor recovery, disrupted corticospinal tract projections, and reduced MAP2 expression. The convergence of behavioral, anatomical, and molecular deficits between neuron‐specific and global *Ttbk2* knockouts supports the interpretation that neuronal *Ttbk2* plays a central role in mediating these effects.

These indicate the significance of TTBK2/PC in neural circuit reorganization after SCI. Therefore, our study originally demonstrates that TTBK2‐mediated PCs play a key regulatory role in neural repair and regeneration after SCI, spanning the three critical phases of SCI repair: neuronal survival, axonal growth, and circuit reconstruction.

This study has several limitations. First, we were unable to resolve TTBK2 and downstream cilium–SHH signaling changes at cell‐type or region‐specific resolution. Although our data suggest neuron‐enriched effects, definitive localization will require spatial transcriptomics or single‐cell mapping. Second, we assessed only partial aspects of ciliary signaling. Direct measurement of Smoothened (Smo) trafficking dynamics—its entry, residence, and exit from the cilium—was not performed, leaving a critical layer of SHH regulation uncharacterized. Finally, downstream mechanisms linking TTBK2‐dependent ciliary signaling to synaptic function and neuronal excitability remain unclear. Future work combining spatial genomics with broader genetic strategies, including glial‐ or cilium‐specific deletions and targeted SHH‐pathway modulation, will be essential to establish a cell‐type–resolved framework for TTBK2‐mediated neurorepair.

## 5 Conclusion

This study systematically elucidates the critical regulatory role of the TTBK2/PC in neural repair and regeneration after SCI. The TTBK2/PC pathway promotes neuronal axonal growth by activating the downstream SHH pathway. In addition, the TTBK2/PC participates in multiple key aspects of endogenous neural regeneration after SCI, including neuronal survival, synaptic excitability, and circuit reconstruction. Our study provides a new mechanistic perspective for understanding repair after CNS injury and informs potential therapeutic targets for neural repair and regeneration following SCI.

## Author Contributions

R.Z. performed all experiments and analyzed the data. S.P., Z.T., H.D., and J.W. helped in modeling, animal behavioral tests, and cellular assays. X.Y. and Z.Q. conceptualized the study, performed analyses, and drafted the manuscript with inputs from all authors. All authors have read and approved the final manuscript.

## Funding

This work was supported by the National Natural Science Foundation of China, 82171388, 82471415.

## Ethics Statement

All animal experiments were conducted per the Guidelines for the Protection and Utilization of Laboratory Animals and approved by the Animal Protection and Utilization Committee of Jilin University (animal ethics protocol number KT202103010; approval date, March 10, 2021).

## Conflicts of Interest

The authors declare no conflicts of interest.

## Supporting information


**Figure S1:** TTBK2 deletion alters expression of cilia‐related genes (related to Figure 2).
Figure **S2** TTBK2 and KIF3A alters expression of KEGG pathway (related to Figure 2).
Figure **S3** Gene–concept networks of axonal and synaptic DEGs in shTTBK2 neurons (related to Figure 2).
Figure **S4** Generation and validation of the *Ttbk2* ^fl/fl^‐Rosa‐Cre ERT^+/−^ mouse model (related to Figure 4).
**Figure S5:** cns70763‐sup‐0001‐FigureS1‐S9.doc. *Ttbk2* ^fl/fl^‐Rosa‐Cre ERT^+/−^ mouse organ changes.
**Figure S6:** Luxol Fast Blue staining result.
Figure **S7** SHH pathway modulates MAP2 expression after SCI.
Figure **S8** Generation and behavioral analysis of the *Ttbk2* ^fl/fl^‐AAV9‐hsyn‐Cre spinal cord hemisection model.
Figure **S9** Neuron‐specific Ttbk2 deletion recapitulates the axonal regeneration and neuronal integrity deficits observed in global knockout mice after SCI.

## Data Availability

The data that support the findings of this study are available on request from the corresponding author. Any additional information required to reanalyze the data reported in this paper is available from the lead contact upon request. GEO: accession number: qrkvgawyvtkjhun and are publicly available as of the date of publication.
