# Supplementary material for: TTBK2‐Driven Ciliogenesis Is Required for Intrinsic Neuronal Regeneration After Spinal Cord Injury
Source: CNS Neurosci Ther. 2026 Jan 24;32(1):e70763. doi: 10.1002/cns.70763 (PMC12831131; doi:10.1002/cns.70763)
Supplement: Supplementary file 1 — Figure S1: TTBK2 deletion alters expression of cilia‐related genes (related to Figure 2). Figure S2 TTBK2 and KIF3A alters expression of KEGG pathway (related to Figure 2). Figure S3 Gene–concept networks of axonal and synaptic DEGs in shTTBK2 neurons (related to Figure 2). Figure S4 Generation and validation of the Ttbk2 fl/fl‐Rosa‐Cre ERT+/− mouse model (related to Figure 4). Figure S5: cns70763‐sup‐0001‐FigureS1‐S9.doc. Ttbk2 fl/fl‐Rosa‐Cre ERT+/− mouse organ changes. Figure S6: Luxol Fast Blue staining result. Figure S7 SHH pathway modulates MAP2 expression after SCI. Figure S8 Generation and behavioral analysis of the Ttbk2 fl/fl‐AAV9‐hsyn‐Cre spinal cord hemisection model. Figure S9 Neuron‐specific Ttbk2 deletion recapitulates the axonal regeneration and neuronal integrity deficits observed in global knockout mice after SCI. [file CNS-32-e70763-s001.doc]

**TTBK2-Driven Ciliogenesis Is Required for Intrinsic Neuronal Regeneration after Spinal Cord Injury**

Renfeng Zhang, Su Pan, Zhenwei Tian, Haorui Du, Jintao Wang, Xiaoyu Yang, and Zhiping Qi

**Figure S1 TTBK2 deletion alters expression of cilia-related genes (related to Figure 2)**

(a–c) Volcano plots illustrating differentially expressed genes between TTBK2-OE vs. NC, TTBK2-OE+shKIF3A vs. NC, and TTBK2-OE+shKIF3A vs. shKIF3A. The x-axis shows log_2_(fold change), and the y-axis shows −log_10_(padj). Blue dots represent non-significant genes (padj > 0.05); green and red dots indicate down- and upregulated genes, respectively.

(d) Heatmap showing gene expression profiles across groups. Color intensity reflects relative expression levels.

(e, f) GO enrichment analysis of TTBK2-OE vs. NC. Upregulated genes were primarily associated with G-protein–coupled receptor activity, whereas genes related to extracellular matrix organization were downregulated. Circle size represents the number of genes per GO term; x-axis indicates the ratio of enriched genes to total genes.

**Figure S2 TTBK2 and KIF3A alters expression of KEGG pathway (related to Figure 2)**

(a) KEGG pathway enrichment analysis indicating that PI3K-Akt, Hedgehog, and calcium signaling pathways are associated with TTBK2 and KIF3A expression.

(b) KEGG pathway analysis between TTBK2-OE and NC groups revealed enrichment in PI3K-Akt and neuroactive ligand–receptor interaction pathways.

(c, d) C presents the KEGG pathway enrichment results that were upregulated in the shKIF3A versus NC comparison; the corresponding downregulated results are shown in D.

(e, f) The KEGG pathways upregulated in the shTTBK2 group compared with the NC group are presented in E, and the downregulated pathways in F.

**Figure S3 Gene–concept networks of axonal and synaptic DEGs in shTTBK2 neurons (related to Figure 2)**

(a) Gene–concept network illustrating interactions between DEGs in the shTTBK2 group and axonogenesis-related GO terms. Node size indicates the degree of connectivity; gene color reflects log₂(fold change), ranging from blue (< 0) to red (> 0). SHH-related genes were among the most highly downregulated.

(b) Network depicting interactions between DEGs and synapse-related GO terms in the shTTBK2 group, using the same color and size coding as in A.

**Figure S4 Generation and validation of the *Ttbk2*^fl/fl^-Rosa-Cre ERT^+/-^ mouse model (related to Figure 4)**

(a) Representative agarose gel electrophoresis results for genotyping of *Ttbk2*^flox/flox^ (left) and Cre ERT^+/-^ (right) alleles. The gel images on the left show genotyping of the Ttbk2 floxed allele. All three candidate mice displayed a single 267-bp band, whereas the wild-type controls exhibited only a 204-bp band, indicating that all three mice were homozygous for the *Ttbk2*^flox^ allele (*Ttbk2*^flox/flox^).The gel images on the right show genotyping of the Cre recombinase allele. Among the three *Ttbk2*^flox/flox^ offspring, one mouse presented a single 446-bp band—distinct from the 967-bp wild-type band—confirming a Cre homozygous genotype (Cre^+/+^). The remaining two *Ttbk2*^flox/flox^ mice lacked the 446-bp band and were identified as Cre^−/−^. (n = 3 mice).

(b) Schematic diagram illustrating the T10 spinal cord hemisection model in mice.
(c) Western blot showing efficient knockout of TTBK2 protein in spinal cord tissues after tamoxifen injection (n = 3 mice).

(d) Illustration of lesion site morphology.

(e) Gross morphological view of the injured spinal cord.No visible differences were observed in the spinal cord tissues of the WT-SCI and *Ttbk2*^fl/fl^-SCI groups.

(f) Schematic diagram of footprint analysis experiment.

(g) Representative images of hindlimb recovery outcomes in SCI mice.

**Figure S5 *Ttbk2*^fl/fl^-Rosa-Cre ERT^+/-^ mouse organ changes**

(a-b) Representative H&E-stained sections of heart, liver, and kidney tissues. *Ttbk2* conditional knockout induced lipid accumulation in liver, with hepatocytes showing cytoplasmic rarefaction and vacuolar changes (blue arrows), and cytoplasmic heterogeneity (green arrows). No significant histological changes were observed in cardiac tissue (n = 3 mice).

Scale bars: 20μm;

T-test was used for statistical analysis; ****P* < 0.001.

**Figure S6 Luxol Fast Blue staining result**

(a) Luxol Fast Blue staining of spinal cord sections reveals myelin distribution. Myelin sheaths appear blue, and nuclei are stained purple. The TTBK2^fl/fl^-SCI group exhibited demyelination (red arrows) compared to controls (n = 3 mice).

Scale bars: upper panel 200 μm, lower panel 50 μm.

**Figure S7 SHH pathway modulates MAP2 expression after SCI**

(a) Western blot showing efficient knockout of TTBK2 protein in spinal cord tissues (n = 3 mice).

(b) Statistical analysis of MAP2 ratio in western blot experiment.

Bars and errors represent mean ± SEM. One-way ANOVA was used for statistical analysis; **P* < 0.05.

**Figure S8 Generation and behavioral analysis of the *Ttbk2*^fl/fl^-AAV9-hsyn-Cre spinal cord hemisection model**

(a) Western blot showing efficient knockout of TTBK2 protein in spinal cord tissues (n = 3 mice).

(b) Statistical analysis of TTBK2 ratio in western blot experiment. Bars and errors represent mean ± SEM. One-way ANOVA was used for statistical analysis.

(c) qRT-PCR analysis confirming TTBK2 gene knockout in *Ttbk2*^fl/fl^*-*AAV9-hsyn-Cre mice (n = 6 from six mice in each group).

(d) BMS revealed significantly better locomotor recovery in NC-SCI mice compared to AAV-Cre-SCI and *Ttbk2*^fl/fl^-SCI mice at 28 days post-injury (n = 9 from nine mice in each group). Two-way ANOVA was used for statistical analysis.

**P* < 0.05, ***P* < 0.01, ****P* < 0.001.

**Figure S9 Neuron-specific Ttbk2 deletion recapitulates the axonal regeneration and neuronal integrity deficits observed in global knockout mice after SCI**

(a, b) BDA tracing showed higher axonal labeling in AAV-Cre-SCI and *Ttbk2*^fl/fl^-SCI mice at the T10 level. However both AAV-Cre-SCI and *Ttbk2*^fl/fl^-SCI mice showed markedly reduced distal BDA labeling (L1 level) relative to NC-SCI mice, indicating impaired long-distance regenerative capacity (*P*<0.01). No significant differences were detected between AAV-Cre-SCI and *Ttbk2*^fl/fl^-SCI groups (*p > 0.05*). Scale bars: left, 200 μm; right, 50 μm.

Scale bars: left, 200 μm; right (Zoom), 50 μm (n = 3 from three mice in each group).

(c) Western blotting analysis of MAP2 expression in spinal cord tissue from NC, NC-SCI, AAV-Cre, AAV-Cre-SCI, *Ttbk2*^fl/fl^, and *Ttbk2*^fl/fl^-SCI mice (n = 3 biological replicates per group). Both AAV-Cre-SCI and *Ttbk2*^fl/fl^-SCI groups showed comparable reductions in MAP2 levels, each significantly lower than NC-SCI.

(d) Quantification of MAP2 protein levels corresponding to (C).

Data are presented as mean ± SEM, analyzed by one-way ANOVA followed by Tukey’s post hoc test.**P*<0.05, ***P* < 0.01, ****P* < 0.001.
